# Accumulation of DNA methylation alterations in paediatric glioma stem cells following fractionated dose irradiation

**DOI:** 10.1186/s13148-020-0817-8

**Published:** 2020-02-11

**Authors:** Anna Danielsson, Kristell Barreau, Teresia Kling, Magnus Tisell, Helena Carén

**Affiliations:** 1 Sahlgrenska Cancer Center, Department of Oncology, Institute of Clinical Sciences, Sahlgrenska Academy, University of Gothenburg, Gothenburg, Sweden; 2 Sahlgrenska Cancer Center, Department of Laboratory Medicine, Institute of Biomedicine, Sahlgrenska Academy, University of Gothenburg, Gothenburg, Sweden; 3 Department of Clinical Neuroscience and Rehabilitation, Institute of Neuroscience and Physiology, Sahlgrenska Academy, University of Gothenburg, Gothenburg, Sweden

**Keywords:** Epigenetics, DNA methylation, Radiation, Paediatric, Glioblastoma, Cancer stem cells, Genomic feature analysis, Repetitive DNA elements, Cancer treatment

## Abstract

**Background:**

Radiation is an important therapeutic tool. However, radiotherapy has the potential to promote co-evolution of genetic and epigenetic changes that can drive tumour heterogeneity, formation of radioresistant cells and tumour relapse. There is a clinical need for a better understanding of DNA methylation alterations that may follow radiotherapy to be able to prevent the development of radiation-resistant cells.

**Methods:**

We examined radiation-induced changes in DNA methylation profiles of paediatric glioma stem cells (GSCs) in vitro. Five GSC cultures were irradiated in vitro with repeated doses of 2 or 4 Gy. Radiation was given in 3 or 15 fractions. DNA methylation profiling using Illumina DNA methylation arrays was performed at 14 days post-radiation. The cellular characteristics were studied in parallel.

**Results:**

Few fractions of radiation did not result in significant accumulation of DNA methylation alterations. However, extended dose fractionations changed DNA methylation profiles and induced thousands of differentially methylated positions, specifically in enhancer regions, sites involved in alternative splicing and in repetitive regions. Radiation induced dose-dependent morphological and proliferative alterations of the cells as a consequence of the radiation exposure.

**Conclusions:**

DNA methylation alterations of sites with regulatory functions in proliferation and differentiation were identified, which may reflect cellular response to radiation stress through epigenetic reprogramming and differentiation cues.

## Background

Paediatric high-grade glioma, including glioblastoma, is one of the most aggressive types of brain tumours. The molecular subtype, determined by DNA methylation profiling, is strongly associated with clinical features [1]. In contrast to adult glioblastoma where most cases have extensive accumulated genetic aberrations, paediatric glioblastoma is characterised by epigenetic deregulation of functions involved in developmental biology [2].

Radiation is an important therapeutic tool for children diagnosed with brain tumours and used as standard treatment for children from the age of 4–5. However, radioresistance remains a major obstacle for radiotherapy treatment [3]. Genetic and epigenetic variabilities, which are currently not fully understood, influence, both directly and indirectly, the cellular response to radiation. Adverse late effects from radiation therapy is common but differ markedly on an individual basis, with one third of patients suffering from severe cognitive disabilities and one third having a seemingly normal development [4].

The biochemical processes that occur within a cell following irradiation are well known, but alterations of the epigenome by radiation have not been thoroughly investigated. Radiation strikes and disrupts the DNA molecule directly by ionization and indirectly by the production of free radicals. Both these actions take place very quickly, in less than a second [5]. However, the biological effects become apparent in hours or days. Ionizing radiation disrupts chemical bonds and impacts specific parts of the DNA molecule, which challenges the cell to continue replication and cell division. If the damage is sufficient to kill the cell, death can take place days or weeks later. Crucial cells as haematopoietic stem cells are the definitive dose-limiting factor of all radiotherapy treatment plans [6]. Still, the dose must be sufficient to eradicate all tumour cells with the potential to repopulate the tumour.

DNA methylation influences phenotypic variation including radiation response. As the cellular radio-response aims to protect the cell, specific DNA methylation alterations may define mechanisms mediating cell survival after radiation exposure and hence constitute a target for radiosensitization [7]. Profiling of altered DNA methylation patterns following irradiation may identify reversible stress adaptations strategies, as cell lineage-specific programs controlled by epigenetic mechanisms could be one way for the cells to resist stress and survive treatment [8, 9]. Cancer stem cells, with their self-renewal capacity and ability to maintain an undifferentiated state, are reported to be particularly resistant against radiation and are thus crucial for brain tumour repopulation and progression [10, 11]. Understanding the cellular and molecular mechanisms of these cancer stem cells involved in the response and resistance to radiotherapy is important to improve therapy.

The overall aim of this study was to identify radiation-induced cytosine methylation alterations; initially (3 FDIR) and after an accumulated methylation drift over repeated radiation dose fractionations (15 FDIR). Identifying targets and dynamics of DNA methylation alterations following radiation has the potential to reveal biological consequences. For this, we used patient-derived paediatric high-grade glioma stem cells (GSCs) grown under conditions that preserve the signatures of the originating tumour and maintain the tumour-initiating capacity of the cells [12]. Genome-wide DNA methylation maps of pre- and post-irradiated GSC cultures were generated to determine the effects radiation has on epigenomic plasticity and stability.

## Methods

### Patients and cell cultures

Cell cultures established from five paediatric patients (three males and two females aged 4–12) with high-grade glioma tumours were used. Cultures originated from pre-radiotherapy tissues except for one that was derived from a tumour relapse post-radiotherapy. All patients received conventional doses of 1.8 Gy in daily fractions (5 days a week) and a total dose of 54 Gy as part of their clinical management. Patients, tumours and cell cultures have been fully described previously [12]. Samples were collected with written informed consent according to the ethical approval (Dnr 604-12). Cells were cultured on laminin-coated (0.5 μg/cm^2^, Sigma-Aldrich, Sweden, or Biolamina, Sundbyberg, Sweden) plastics (Costar, Washington) in stem cell media (DMEM-F12 supplemented with B27 (Gibco, Thermo Fisher, Waltham, Massachusetts), N2 (Gibco) and EGF (10 ng/ml, Peprotech, Stockholm, Sweden)). Fresh media was replaced every fourth day, and cells were passaged typically every week, 1:2–1:5, using Accutase (Gibco). All established cell cultures were confirmed negative for mycoplasma contamination.

### Irradiation

**Fig. 1.**
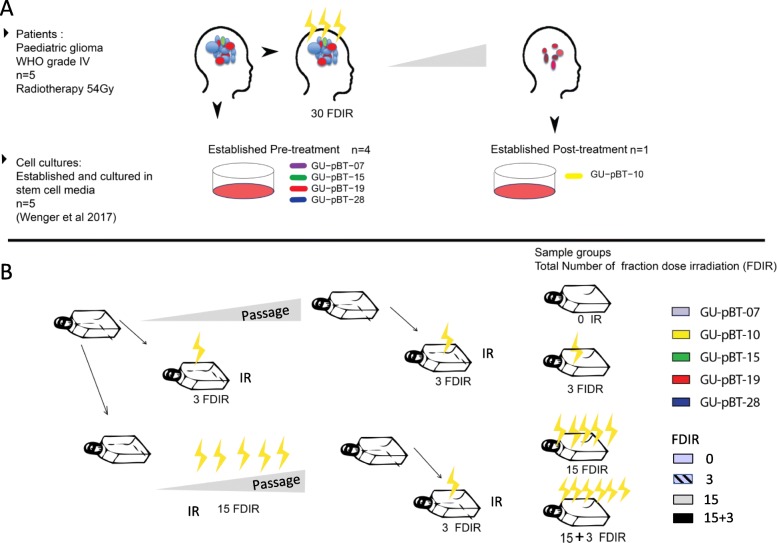
Schematic representation of the model system and experimental setup of fractionated dose irradiation schemes. **a** Paediatric high-grade glioma stem cell cultures established pre- or post-radiotherapy (30 fractions irradiation of 1.8 Gy each) were used in the study. **b** Cells were exposed to 3–18 fractionated dose irradiations 2–8 Gy. To monitor the stability during culturing, GU-pBT-07 cells were cultured and analysed at passage 12 and 22. These sub-cultured cells from early (p12) and later passages (p22) were irradiated and used as biological replicates

GSCs were irradiated with repeated doses of 2, 4 or 8 Gy (Fig. 1) at a dose rate of 1.7 Gy/min delivered by a RS 2000 Biological Research Irradiator (Rad Source Technologies, Buford, GA). A 0.3-mm Cu filter for low energy radiation filtration at 160 kV × 25 mA was used. As controls, unirradiated cells from the corresponding passages were handled outside the cell incubator in parallel with irradiated cells. The dose and number of fractions of radiation treatment in this study were determined by proliferation assays and DMP analysis based on preliminary experiments. The aim was to analyse cells that resume cell cycle progression following radiation and to avoid early non-stable DNA methylation effects and early cell death that lack interest for the generation of radiation-resistant phenotypes. Dose-response experiments were performed initially, and 2 Gy was chosen as the dose presented if not otherwise specified. Cells were irradiated for 3 or 15 numbers of fraction dose irradiation (FDIR). The cells that recovered after 15 FDIR were re-irradiated for another 3 fractions (15 + 3) to study this specific population of cells. Cells were assayed at either 72 h, 14 days or 21 days post-radiation.

### Cell viability and cellular characterisation

Morphological features were monitored during culturing and at passaging, comparing radiation-exposed cells with control cells. Representative images were captured over time with an inverted microscope equipped with phase-contrast optics (Leica) using a black and white CCD video camera (Lumenera, Ottawa, Ontario, Canada). The Via1-Cassette™ was used together with the NucleoCounter® NC-3000™ for determination of cell count, cell size and viability. For adherent cells, the LIVE/DEAD® Cell Imaging Kit (Invitrogen) was used to identify cells with intact or permeable membranes. Hoechst (1:2000; Thermo Fisher) was used as a counterstain for cell nuclei. Images were acquired with Operetta High-Content Imaging System, and data processing was performed using the Harmony software version 4.1 (Perkin Elmer, Waltham, Massachusetts). FDIR cells and the corresponding unirradiated cell population were analysed for phenotypic growth pattern and expression of stem cell markers with immunocytochemistry (ICC) method. Cells were seeded in 96-well plates. Proliferation was assessed with the Click-iT EdU AlexaFluor 488 Imaging Kit (C10337, Thermo Fisher, Waltham, Massachusetts). Briefly, cells were incubated during 4 or 24 h with EdU (5 μmol/L final concentration) prior to fixation with paraformaldehyde 4% (Histolab, Västra Frölunda, Sweden), in phosphate-buffered saline (PBS, 0.1 M, pH 7.4) for 10 min, at 14 days after irradiation. After washing quickly twice and once for 30 min with PBS, the cells were permeabilised 30 min with PBST (PBS with 0.1% Triton X-100). Cells were washed once with PBST and incubated with block solution (3% goat serum (Fisher Scientific, Hampton, New Hampshire) and 1% BSA (bovine serum albumin; Sigma, Saint-Louis, Missouri) in PBS for 1 h at room temperature. Primary antibodies used were as follows: mouse anti-α-tubulin (1/1000; T6199, Sigma) and anti-GFAP (1/1000; G3893, Sigma). All primary antibodies were diluted in block solution. The cells were washed with PBS and incubated 1 h at room temperature with the secondary antibodies. Secondary antibodies were chosen according to the primary antibodies: AlexaFluor594-coupled anti-mouse antibody (A21125, Invitrogen, Carlsbad, CA), AlexaFluor647-coupled anti-rabbit antibody (A121244, Invitrogen) and AlexaFluor594-coupled anti-rabbit antibody (A11012, Invitrogen). All secondary antibodies were diluted at 1/1000 in block solution or fluorescence antibody diluent (FAD; FAD901L, Biocare Medical, Pacheco, CA) for the OLIG2 secondary antibody. Cells were washed with PBS and counterstained with DAPI (1/5000; D9542, Sigma) in PBS for 5 min.

### DNA sample preparation and DNA methylation profiling

DNA was extracted using the DNeasy Blood & Tissue Kit (Qiagen, Hilden, Germany) according to the protocol provided by the supplier. The DNA was quantified with a Qubit fluorometer (Life Technologies, Carlsbad, CA), and 500 ng was used for bisulfite modification with the EZ DNA methylation kit (D5001, Zymo Research, Orange, CA). The bisulfite-modified DNA was applied to the Infinium HumanMethylation450 BeadChips (Illumina, San Diego, CA) which determine the methylation levels of more than 450,000 CpG sites. The fluorescence signals were measured from the BeadArrays using an Illumina BeadStation GX scanner and further processed with Bead studio to obtain raw data for further downstream analysis.

### Data and statistical analysis

Array data were analysed using the R package ChAMP [13]. Probes with multiple and nonspecific mapping from their intended target were removed before normalisation. Each CpG site was assigned a score called a “β-value”, which corresponds to the ratio between the fluorescence signal of the methylated allele (C) and the sum of the fluorescent signals of the methylated (C) and unmethylated (T) alleles. DNA methylation levels were depicted as beta values in the interval 0–1. Differential methylation with delta beta below 0.2 was omitted before further analysis. Chromosome coordinates refer to human genome assembly hg19. Methylation age was determined with Horvath age [14]. The GISTIC module was used to identify regions of the genome that were significantly amplified or deleted (ftp://ftp.broadinstitute.org/.../GISTIC2*).* Graphs were made in R, Pandas Seaborn and Graph Pad. The Gene Set Enrichment Analysis (GSEA) software was used to test for the over-representation of Gene Ontology (GO) biological processes and molecular functions (http://www.geneontology.org/). Annotation of Infinium DNA Methylation BeadChip probes was provided by the HumanMethylation450 v1.2 Manifest.

The R package Repetitive Element Methylation Prediction (REMP) version 1.10.0 [15] was used to calculate methylation β-values of Alu elements.

## Results

In this study, we profiled DNA methylation alterations that follow after few (*n* = 3) and many (*n* = 15 or 15 + 3) fractions of radiation. For this, we used previously generated GSC cultures [12] established from patients before (*n* = 4) and after treatment (*n* = 1). These well-characterised cells have been shown to be stable during culturing and maintain their DNA methylation profiles and DNA copy number alterations (CNA). The expression of stem cell proteins as well as in vivo tumour-initiating capacity of these cells [16] demonstrates their resemblance to cancer stem cells or progenitor cells. These cells hence provide a relevant and robust model system for functional studies on paediatric brain tumours. The overall scheme of the study is depicted in Fig. 1.

### Proliferation is reduced and cellular morphology is altered following radiation

**Fig. 2:**
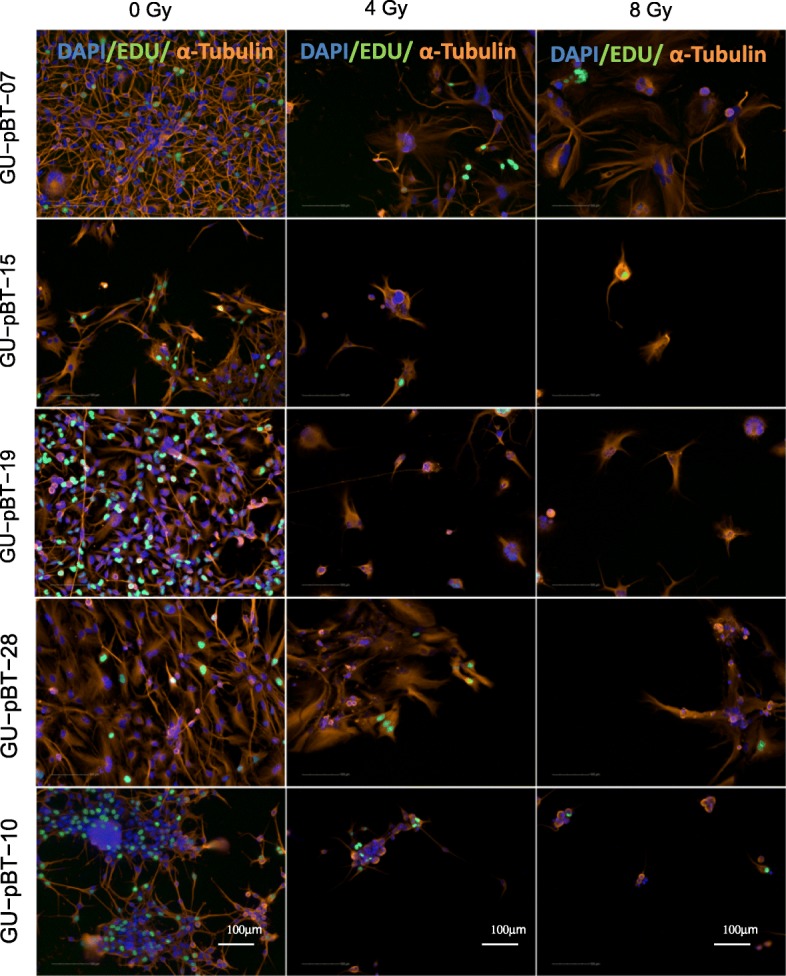
Radiation-induced cellular and nuclear morphological changes. Immunocytochemistry showing cellular morphology with α-tubulin-stained cytoskeleton (orange), DNA-synthesizing cells by EdU incorporation (green) and nuclear staining with DAPI (blue) at normal culture conditions (0 Gy) and exposed to radiation (4 and 8 Gy). Scale bars 100 μm

We first determined whether irradiation changed the cellular morphology of the cells using immunocytochemistry (ICC; Fig. 2). An exponential reduction in cell number as well as alterations in the morphology of the remaining cells (reshaped nuclei, increased polyploidy and senescence morphology) was seen with increasing doses of irradiation. The proportion of cells with active replication, estimated with EdU incorporation, indicated that 2- and 4-Gy-treated cells retained proliferative capacity, while only a small proportion of 8-Gy-treated cells were actively proliferating 72 h post-IR (Fig. 2). In all but one GSC culture, the number of cells with enlarged nuclei and altered morphology was increased (Fig. 2). Surviving cells were able to establish cell colonies when single cells were seeded, demonstrating that the cells maintained the capacity to repopulate (data not shown).

### Short-term doses of radiation do not alter genome-wide DNA methylation levels of GSCs

**Fig. 3:**
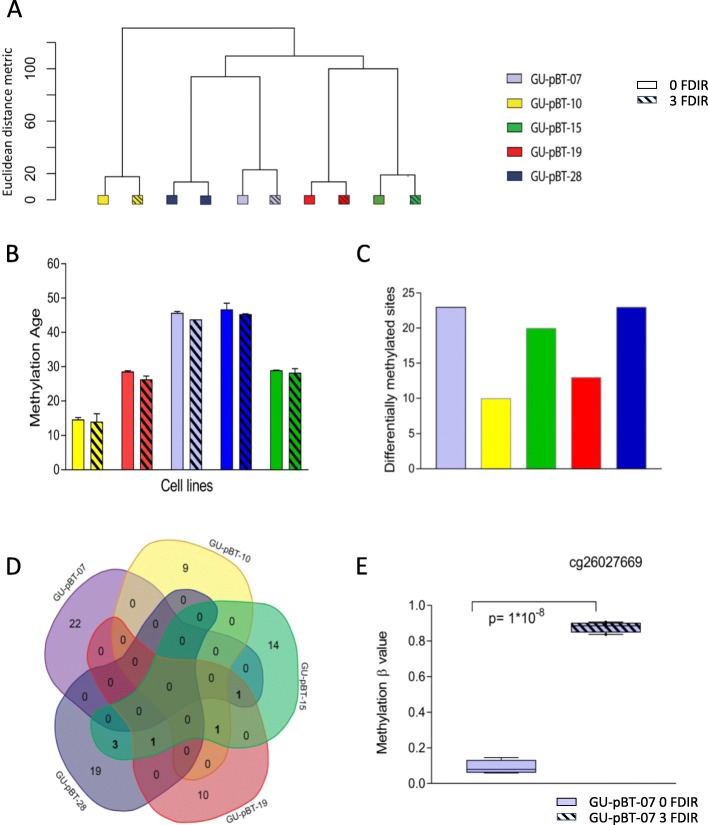
Genome-wide and site-specific methylation alterations following three FDIR of 2 Gy. **a** Unsupervised clustering and **b** DNA methylation age calculated with Horvath’s DNA methylation age calculator show that cells maintain their DNA methylation profiles of specific sites with radiation. Error bars represent standard error of mean, *n* = 2. **c** Number of differentially methylated positions (DMPs) detected in each cell line. **d** Venn diagrams depicting the overlapping DMPs between the cell lines. **e** A selected CpG site showing a change from unmethylated to methylated state following radiation at both early and late passages and at both 2 and 4 Gy

We next profiled DNA methylation of cells following three fractions of radiation to assess the DNA methylation instability. Supervised hierarchical clustering of the top 1000 most variable sites showed that the individual GCS cultures clustered separately, independent of the irradiation treatment, demonstrating that the genome-wide methylation profiles did not alter substantially (Fig. 3a). DNA methylation can be used to accurately predict biological age. As the predicted age can be altered by external stimuli, we analysed whether the methylation age of the cells was altered with radiation. For this, we used the Horvath’s epigenetic clock, a tissue-independent clock that aims to predict chronological age using methylation data [14]. We detected very small alterations in the predicted DNA methylation age following three FDIR (Fig. 3b).

### Few site-specific DNA methylation alterations following three irradiation fractions

Since the global profile was stable, we next analysed whether there were site-specific alterations with radiation. For this, we identified the number of differentially methylated positions (DMPs) that occurred with radiation for the five GCS cultures respectively (Fig. 3c). In all cases, a low number of DMPs were identified (*n* = 10–23). Few of these were detected in several of the individual GSC cultures (Fig. 3d). One of the identified DMPs, cg26027669, in the growth factor independent 1 transcriptional repressor gene (*GFI1*), showed large, consistent alterations in methylation frequency with both 2 and 4 Gy radiation in one of the GSC cultures, GU-pBT-07 (Fig. 3e). This site was unmethylated in the unirradiated cultures (methylation beta value < 20%) and highly methylated (> 80%) in the irradiated cultures and could hence constitute a tumour-specific marker of radiation exposure, independent of dose. To validate this alteration, we repeated the experiment using cells at a higher passage which identified the same DNA methylation alteration.

### Extended dose fractionations alter DNA methylation profiles and induce thousands of DMPs

**Fig. 4:**
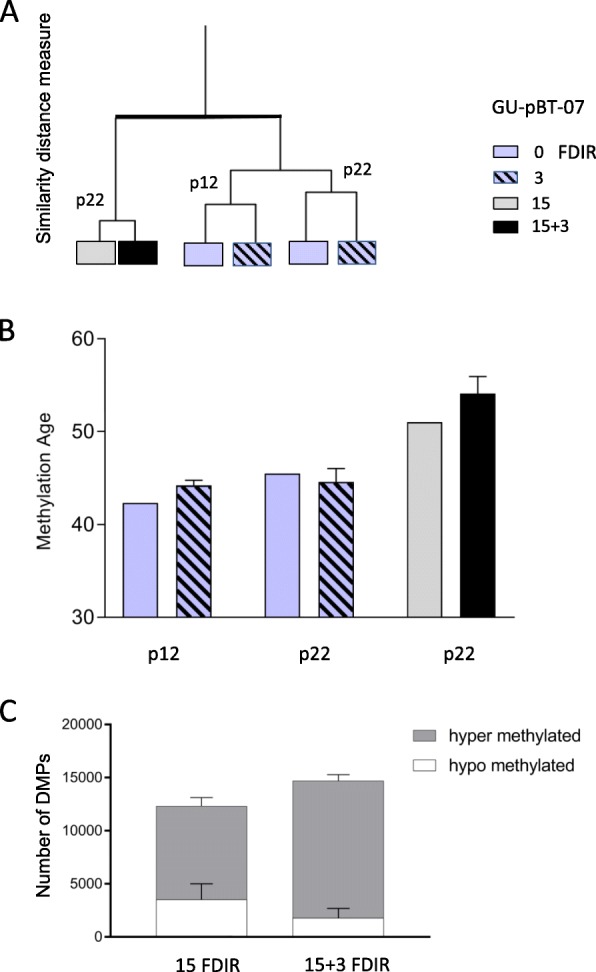
Alterations in DNA methylation levels following 3, 15 and 15 + 3 fractional doses. **a** Unsupervised clustering of methylation beta values. **b** DNA methylation age. **c** Number of hypomethylated (white) and hypermethylated (grey) DMPs observed in the IR samples compared with unirradiated samples. Error bars represent standard error of mean. To address the comparability of the cells between different passages, experiments were performed at both mid and late passages for both control and 3 FDIR

As we detected few alterations, and to more mimic the clinical situation, we irradiated the most radioresistant GSC culture GU-pBT-07, with repeated fractions (*n* = 15). The 15 FDIR treatment was used to evaluate the population of cells that survived and repopulated during treatment. These cells were then irradiated for another three fractions (15 + 3) to study the response of the radioresistant cells. The change in DNA methylation profile following repeated fractions of irradiation was evident when the data was visualised in an unsupervised clustering (Fig. 4a). DNA methylation age calculations based on Horvath’s tissue independent CpG sites showed accelerated age following repeated irradiation fractions (Fig. 4b). We next analysed DMPs following the repeated fractions of 2 Gy irradiation and detected a 1000-fold increase in the number of sites compared to the three fraction set up (Fig. 4c). Comparing the number of DMPs between the 15 and 15 + 3 fractions showed that the majority of sites were consistently detected in both treatment groups (data not shown). The DMPs with the largest difference in methylation value (delta beta) are presented in Additional file 1: Table S1. To determine whether the shifted DNA methylation profiles following 15 and 15 + 3 FDIR was a consequence of the radiation exposure and not general cell culture artefacts, we cultured unirradiated cells for the same time as the duration of the irradiations. This resulted in very few changes, demonstrating that the alterations following 15 and 15 + 3 fractions were indeed due to the irradiation and not a culture artefact (data not shown). In addition, we inferred chromosomal copy number status from the array data which verified the chromosomal stability of the cells during the experiment (Additional file 2: Figure S1).

### The differentially methylated positions are enriched in enhancer and R-DMR regions

**Fig. 5:**
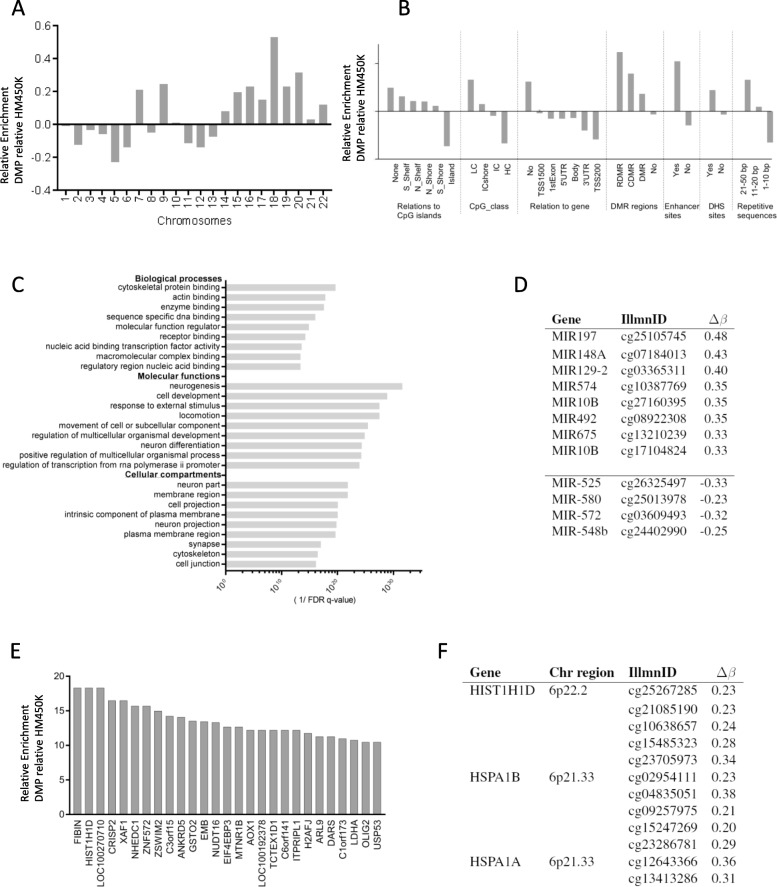
Distribution of DMPs in unirradiated versus 15 + 3 FDIR samples. **a** Chromosomal location. **b** Genomic features. **c** Gene Ontology enrichment analysis (cellular compartments, molecular functions and biological processes). **d** hsa-miR genes. **e** Genes with higher number of DMP than expected. **f** Differentially methylated regions (DMRs) implicated in known radiation response functions

By analysing the chromosomal distribution of the DMPs following 15 and 15 + 3 FDIR, we detected enrichment at specific chromosomes, for example at chromosome 18 (Fig. 5a). The location of the DMPs was enriched in enhancer regions, regions of repetitive DNA elements and in regions previously demonstrated to be differentially methylated in cancer (C-DMRs) and during reprogramming (R-DMRs) [17] (Fig. 5b). Less sites, in relation to the representation of sites on the array, were detected in the CpG islands and around the transcription start sites. We also noted an overrepresentation of sites annotated as involved in alternative splicing among the identified DMPs (1.5-fold enrichment, BH-corrected *p* value of 9.7 × 10^−4^). Pathway analysis using the DMPs identified relevant terms such as neurogenesis, development and cell differentiation (Fig. 5c).

MicroRNAs (miRNAs) typically repress gene expression, and modulation of specific miRNAs may provide therapeutic targets. The methylation arrays include 35,699 CpG sites annotated to the promoter regions of non-coding RNA genes. The majority of the 82 identified DMPs in regions associated with miRNAs increased in methylation following radiation, and only eight showed decreased methylation levels. Several miRNAs that have previously been related to stem cells, invasion, migration and radiation response were identified as altered (Fig. 5d). The top hypomethylated miRNA, after irradiation, was miR-525. This miRNA has previously been demonstrated to be upregulated in response to radiation [18].

We further analysed genes enriched with DMPs and found among these, for example, two histone genes (*HIST1H1D* and *H2AFJ*), a gene involved in DNA damage repair (*NHEDC1*) and a gene in stem cell regulation (*OLIG2*) (Fig. 5e). Moreover, two HSP70 heat shock protein genes, *HSPA1A* and *HSPA1B*, were identified (Fig. 5f). Among the 100 CpG sites with the largest radiation-induced difference in methylation, three sites in the gene *SHROOM3*, encoding for the actin-binding protein and cell morphology regulator, were identified (Additional file 1: Table S1). The Sphingosine-1-Phosphate Transporter (SP1) gene *MFSD2B*, a regulator of self-renewal and differentiation in neural and cancer progenitor cells [19], was also differentially methylated at multiple CpG sites (*n* = 6). Other genes with many altered sites included the glial fibrillary acidic protein *GFAP*, which encodes one of the major intermediate filament proteins of mature astrocytes, and the glutathione metabolism gene *GSTP1.*

### Frequent hypomethylation of repetitive elements occurs following radiation

The majority of methylated CpG sites in the genome are located within regions of repetitive DNA elements, and we therefore specifically analysed probes in these regions. Methylation β-values of repetitive Alu elements were predicted using the R package REMP. These analyses identified global hypomethylation in Alu elements following irradiation (Additional file 2: Figure S2A). The Pearson correlation in methylation beta values between untreated cells at different passages, as well as between untreated cells and cells irradiated with three fractions, was high (0.99). Following 15 or 15 + 3 fractions, the correlation decreased with an increased number of repetitive base pairs in the probe sequence down to 0.95 (Additional file 2: Figure S2B). This indicates that there is an increased methylation variability with an increasing number of repetitive base pairs. Our results thus confirm previous studies showing that irradiation results in hypomethylation of repetitive elements [20].

### Fractionated irradiation alters cellular properties

**Fig. 6:**
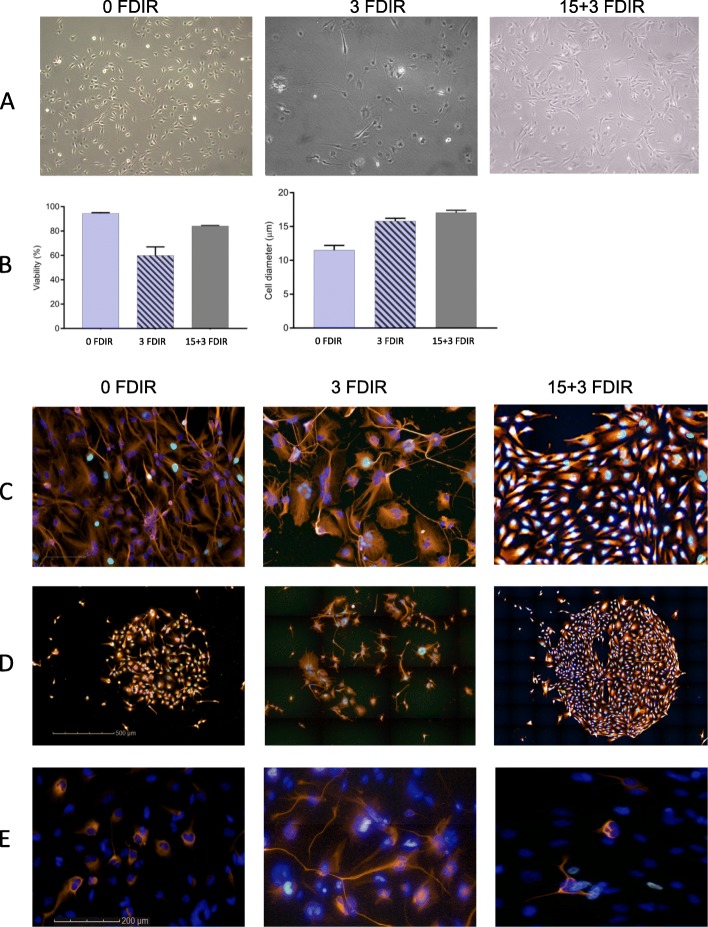
Radiation-induced morphological changes with increasing number of radiation fractions of 2 Gy. **a** Bright-field enlarged cells. **b** Percent of viable cells and cell diameter. **c** Immunocytochemistry showing cellular morphology with α-tubulin-stained cytoskeleton (orange), DNA-synthesizing cells by EdU incorporation (green) and nuclear staining with DAPI (blue). **d** Single-cell seeding after irradiation shows reduced clonogenic capacity after 3 fractions compared to unirradiated and 15 + 3 irradiated cells. **e** The frequency and staining pattern of GFAP varied between treatment groups

Cell states are determined by DNA methylation patterns, and we thus explored if the large alterations in the methylome that was induced with fractionated irradiation affected the characteristics of the cells. Irradiation resulted in a higher resistance of the radiation-enriched cells following 15 + 3 fractions compared to 3 fractions (Fig. 6a), and both 3 and 15 + 3 irradiated cells showed increased cell diameter (Fig. 6a, b). Extensive alterations between unirradiated cells and cells irradiated with 3 or 15 + 3 fractions were detected, including reorganisation of the cytoskeleton, altered proliferation rate and growth patterns (Fig. 6c). Colonies that formed after single-cell seeding showed an inter-population variation but a high homogeneity within colonies in terms of cellular morphology, proliferation rate and expression of neural and progenitor cell marker proteins (Fig. 6d). The colony-forming ability following 21 days after irradiation decreased after 3 fractions; however, colonies obtained from radiation-enriched cells (15 + 3 fractions) were dense and larger in size compared to unirradiated cells (Fig. 6d).

As we detected methylation alterations that could be associated with alternative splicing of the astrocytic gene *GFAP*, we further evaluated this finding on the protein level using ICC. GFAP was distributed more evenly in the cytoplasm after irradiation (Fig. 6e). This pattern has previously been linked to the different isoforms of GFAP [21, 22], suggesting that the differential methylation that we detected could be involved in the regulation.

## Discussion

Cellular response to radiation and intrinsic radiosensitivity are highly influenced by the function of tumour suppressor genes, often epigenetically silenced in tumours [23]. In this study, we therefore aimed to identify loci of DNA methylation alterations in paediatric GSCs following radiation in order to reveal alterations in sites or regions which could mediate the effects of radiation.

The effects of irradiation on gene expression have been documented in some studies, however resulting in very few consistent and informative clinical useful findings [24, 25]. In addition, discrepancy between protein levels and number of RNA molecules is frequently observed [26]. DNA methylation, on the other hand, is a stable mechanism which specifies cell identity and shows tumour entity-specific profiles. DNA methylation response following radiation treatment has previously been reported but not for stem cells or paediatric tumour cells [27–29]. DNA methylation in prostate cancer cells was recently reported to be stable following a single dose of irradiation with few stably altered CpG sites up to 14 days following exposure [30].

Paediatric high-grade gliomas originate from cells along the neural stem cell lineages [31]. These cells have the capacity to generate a variety of different cell types: activated stem cells, progenitor cells and mature cells such as astrocytes, oligodendrocytes and neurons [32]. The DNA methylation pattern is strictly regulated during differentiation and development allowing each cell type to acquire its required phenotype to meet its specific functions and needs. In this study, we used patient-derived GSCs to better reflect the plasticity of cells seen within tumours in vivo. We started with three fractions of irradiation which identified few altered loci (DMPs; > 20% difference in methylation). However, individual DMPs with large alterations could be consistently identified. For example, a CpG site in the gene *GFI1* showed a large difference in methylation level. The change from unmethylated to methylated indicates that the majority of cells altered methylation status at this site following radiation. This alteration was consistently found in several experiments and with different doses of irradiation and could therefore be of interest for elucidating cell type-specific early radiation responses. The methylation state of this site has previously been shown to alter in a leukaemia cell line undergoing differentiation [33].

To more mimic the clinical treatment protocols, we next performed many fractions (15 or 15 + 3) of irradiation, which resulted in large DNA methylation alterations, not detected in unirradiated cells cultured in parallel experiments. Genome-wide alterations, an accelerated DNA methylation age and a high number of detected DMPs (~ 10,000), most of which were hypermethylated, were detected. The genomic regions that were most frequently targets of the detected radiation-induced DNA methylation alterations were regions previously described to be altered in reprogramming (R-DMRs), cancer (C-DMRs) and in enhancers [17]. Alterations were also detected in CpG island shores, regions known to be variable across cell types and variably methylated during cell differentiation [34]. Regions with fewer alterations than expected included promoter regions and CpG islands.

Inhibition of specific miRNAs has been shown to contribute to radiation resistance in various cancer cells and to control the balance between proliferation and differentiation following irradiation [35]. The miR-525 contained the most hypomethylated site among the miRNA sites annotated on the array. Inhibition of miR-525-3p has previously been shown to elevate radiosensitivity, while overexpression of the precursor miR-525-3p conferred radioresistance in endothelial-like cells and various tumour cell lines [18]. Two DMPs were located within miR-10b, and previous studies have shown that high gene expression of this miRNA is associated with disease progression and self-renewal [36]. Functional studies have suggested that miR-10b may function as a tumour suppressor and is silenced by DNA hypermethylation in gastric cancer [37].

Apart from alterations in miRNAs, we also detected many DMPs in protein-coding genes. Several of these were located in the genomic regions of the two histone genes *H2AFJ* and *HIST1H1D*. Histone variant H2A.J accumulates in senescent cells and promotes inflammatory gene expression [38] which implies that the DNA methylation alterations could affect cell fate. The linker histone *HIST1H1D* interacts with linker DNA between nucleosomes and functions in the compaction of chromatin into higher order structures [39]. We additionally identified many altered sites with large delta beta in the Heat Shock Protein Family A (Hsp70) Member 1A and 1B. Previous studies have shown that the *HSPA1A* promoter is methylated in cell lines with low gene expression of *HSPA1A* with expression being restored following treatment with the DNA demethylating agent 5-aza-2′-deoxycytidine [40]. Since heat shock proteins are an attractive target for anticancer therapy, further studies are needed. Also in the SP1 gene *MFSD2B*, many DMPs were identified. SP1 was recently shown to promote tumour growth by inhibiting apoptosis and conferring chemo- and radiation resistance to cancer cells [41]. Furthermore, radiation treatment was found to recruit SP1 transcription factor in meningioma cells [42]. We also detected promoter hypermethylation of the glutathione metabolism gene *GSTP1* in the irradiated cells which is in agreement with a previous study on irradiated leukocytes [43]. The alterations that we identified in these genes could potentially confer adaptation to radio-induced stress.

Knowledge generated from recent studies indicates that changes in DNA methylation in cancer cells impact the organisation of the genome in the nucleus rather than directly affecting gene expression [44]. Repetitive elements represent a large portion of the human genome. DNA methylation, together with other chromatin modifications, is most often associated with silencing of transposable elements (TEs) within repetitive regions [45]. The Alu element is commonly found in repetitive regions. Recently, many environmental factors were shown to alter Alu methylation, which could lead to chromosomal instability resulting in chromosomal rearrangements [46]. We found that loci in long repetitive regions were significantly enhanced in DMPs following 15 and 15 + 3 FDIR, demonstrating that also irradiation can induce such alterations. This is in agreement with earlier studies showing decreased DNA methylation in repetitive elements with radiation in various models [47–50].

To link the DNA methylation findings to cellular properties, we studied the cells with ICC. Altered morphology following radiation was apparent, and biological processes like apical cytoskeletal protein processes within membrane regions were enriched among radiation-induced DMPs. Cells following many fractions of irradiation formed dense colonies with altered morphology in contrast to few fractions where the opposite was seen.

In this study, we could not distinguish between if the identified alterations were due to a selection of cells with specific profiles or to altered DNA methylation levels in cells, since the DNA methylation arrays cannot be performed on individual cells. The irradiation induced methylation alterations, with the majority below 30%, indicating selection of cells with specific profiles. However, sites with large alterations, as were identified for example in the *GFI1* gene, are more likely to have resulted from an active methylation alteration of the cells, due to the short time of the acquired alterations. The alterations could theoretically also result from a genetic instability following radiation exposure. The analysis of CNAs did however not detect relative increase or decrease in DNA copy levels, demonstrating that the genome was stable during the experiments.

## Conclusions

On the epigenetic level, at least three categories of elements were identified as associated with long-term radiation exposure: (1) repetitive sequences, (2) enhancers and (3) sites that distinguish cell types and differentiation states. Although the identification of changes in DNA methylation levels does not prove functional consequences, it demonstrates the need for additional studies that focus on detecting possible functional implications of such accumulation of alterations. The alterations that accompanied the generated radioresistant cells were stable and mark the cell identity and characteristics of these cells. Cellular characteristics showed growth selection from typical mixed morphology to more homogeneous populations.

Identifying DNA methylation marks that drive radiation-induced changes in cell state provides candidates that can potentially be targeted therapeutically. This would offer a way to interfere with the generation of the radiation-resistant phenotype.

## Supplementary information

**Additional file 1: Table S1**. The DMPs with the largest difference in methylation value.

**Additional file 2: Figure S1**. CNA of potential tumour driver and tumour suppressor genes. **Figure S2**. Alterations in DNA methylation of CpG sites associated with repetitive sequences following 15 and 15+3 FDIR of the cell line GU-pBT-07. (a) DNA methylation beta values of Alu elements decreased following 15 and 15+3 FDIR compared to unirradiated and 3 FDIR; (b) Pearson correlation between unirradiated and irradiated cells decreased with increased number of repetitive bp within the probe sequences.

## Data Availability

Data sets will be accessible in GEO upon acceptance of this manuscript.

## Abbreviations

Alu: Arthrobacter luteus
B27: Gibco B-27 Serum Free Supplement
BH: Benjamini and Hochberg procedure
CCD: Charge-coupled device
CpG: Regions of DNA where a cytosine nucleotide is followed by a guanine nucleotide
Cu: Copper
DMEM: Dulbecco’s modified Eagle medium
DMP: Differentially methylated positions
DMR: Differentially methylated regions
EdU: 5-Ethynyl-2′-deoxyuridine
EGF: Epidermal growth factor
F12: Ham’s F-12 nutrient mixture
FDIR: Fractionated dose irradiation
GSC: Glioma stem cells
Gy: Gray
PBS: Phosphate-buffered saline
