# Supplementary material for: Accumulation of DNA methylation alterations in paediatric glioma stem cells following fractionated dose irradiation
Source: Clin Epigenetics. 2020 Feb 11;12:26. doi: 10.1186/s13148-020-0817-8 (PMC7014676; doi:10.1186/s13148-020-0817-8)
Supplement: Supplementary file 1 — Additional file 1: Table S1. The DMPs with the largest difference in methylation value. [file 13148_2020_817_MOESM1_ESM.pdf]

Suppl table 1

IllmnID	Gene	$\Delta\beta$
cg00524708	PRSS36	0.67
cg16219246		0.64
cg00051595		0.64
cg20311846	SHROOM3	0.63
cg10967023		0.61
cg27417997	SHROOM3	0.60
cg05851730		0.60
cg03561565	KCNS2	0.58
cg15046481		0.58
cg03256780	SLC8A1	0.56
cg04955116		0.56
cg00615941		0.55
cg19008222		0.55
cg13338137		0.54
cg05654008	ITGA9	0.54
cg02687055	MFSD2B	0.54
cg06805940	ATP10B	0.54
cg10282845	PPTC7	0.53
cg03344693		0.53
cg16026813	BTRC	0.53
cg23341089		0.53
cg27242549	ZC3H8	0.53
cg27281765	PMP2	0.52
cg06282270	VWCE	0.52
cg04044188	PDSS1	0.52
cg14979674	HCN1	0.52
cg09550083	MEIS1	0.52
cg14283978		0.52
cg27146152	XAF1	0.52
cg04052934		0.52
cg12725323	LCE1D	0.52
cg19162496	SHROOM3	0.52
cg13869401		0.52
cg05964600	HCN1	0.51
cg08759041	MCC	0.51
cg26876444	LEPR	0.51
cg27570256	LOC100270710	0.51
cg15078838	BRE	0.51

IllmnID	Gene	$\Delta\beta$
cg00805867	PDE4B	0.51
cg04464085		0.50
cg25345746		0.50
cg06282029		0.50
cg20924825		0.50
cg17315500		0.50
cg16621833	VGLL2;VGLL2	0.50
cg12280150	HEPHL1	0.50
cg07893738		0.50
cg02360367	SKI	0.50
cg06550214	RNF220	0.50
cg14047478	NTN1	0.49
cg06115810	PDE4B	0.49
cg10116443	SLC22A9	0.49
cg04403415	NCOR2;NCOR2	0.49
cg19130189	MMP16	0.49
cg16320249		0.49
cg12072178	VAMP3	0.49
cg00728890		0.49
cg03489382	CCDC136	0.49
cg12082609	MEIS1	0.49
cg08942800	CRISP2	0.49
cg21832148		0.48
cg15193473	BIVM;BIVM	0.48
cg06339924	TRIM42	0.48
cg10543574		0.48
cg25105745	MIR197	0.48
cg22448304		0.48
cg23737061	FHIT;FHIT	0.48
cg03397031	OPRD1	0.48
cg12109823		0.48
cg25340973	SUCLA2	0.48
cg14326671	LOC100192378	0.48
cg23166389	LHX8	0.48
cg02706919		0.48
cg09347495	CLIC5	0.48
cg04553838	TRPC6	0.48
cg24473633	DENND3	0.48
cg13942283	RBM20	0.47
cg14873600		0.47
cg23490638		0.47
cg06823060	CMIP	0.47
cg19555986	MAP7	0.47
cg17994569	CHAT	0.47

IllmnID	Gene	$\Delta\beta$
cg07102913		0.47
cg24701270	ZNF662	0.47
cg08767938		0.47
cg14257199		0.47
cg20477005	SLC26A10	0.47
cg17663774		0.47
cg13849454		0.47
cg18127012		0.47
cg11367259		0.47
cg17078190		0.47
cg13636952		0.47
cg23273897	MME	0.47
cg09038914	GFAP	0.46
cg15410903	GSTO2	0.46
cg02055483	NPAS4	0.46
cg03122427		0.46
cg01491071		0.46
cg07891120		0.46
