# Supplementary material for: Accumulation of DNA methylation alterations in paediatric glioma stem cells following fractionated dose irradiation
Source: Clin Epigenetics. 2020 Feb 11;12:26. doi: 10.1186/s13148-020-0817-8 (PMC7014676; doi:10.1186/s13148-020-0817-8)
Supplement: Supplementary file 2 — Additional file 2: Figure S1. CNA of potential tumour driver and tumour suppressor genes. Figure S2. Alterations in DNA methylation of CpG sites associated with repetitive sequences following 15 and 15+3 FDIR of the cell line GU-pBT-07. (a) DNA methylation beta values of Alu elements decreased following 15 and 15+3 FDIR compared to unirradiated and 3 FDIR; (b) Pearson correlation between unirradiated and irradiated cells decreased with increased number of repetitive bp within the probe sequences. [file 13148_2020_817_MOESM2_ESM.pptx]

## Slide 1
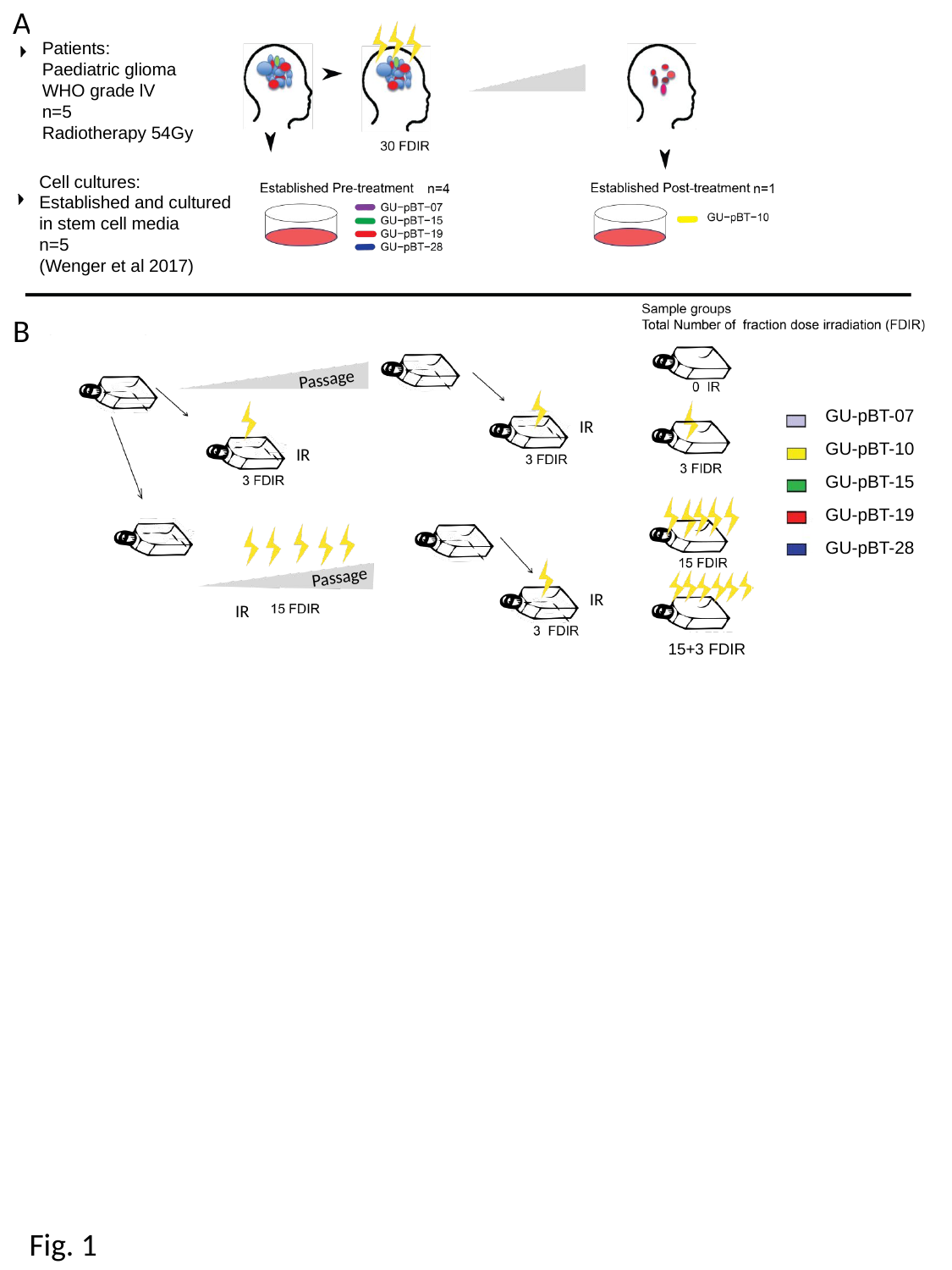

A
B
Passage
IR
IR
Passage
IR
IR
Patients:
Paediatric glioma
WHO grade lV
n=5
Radiotherapy 54Gy
Cell cultures:
Established and cultured
in stem cell media
n=5
(Wenger et al 2017)
GU-pBT-07
GU-pBT-10
GU-pBT-15
GU-pBT-19
GU-pBT-28
 FDIR
0
3
15
15+3
15+3 FDIR
Fig. 1

## Slide 2
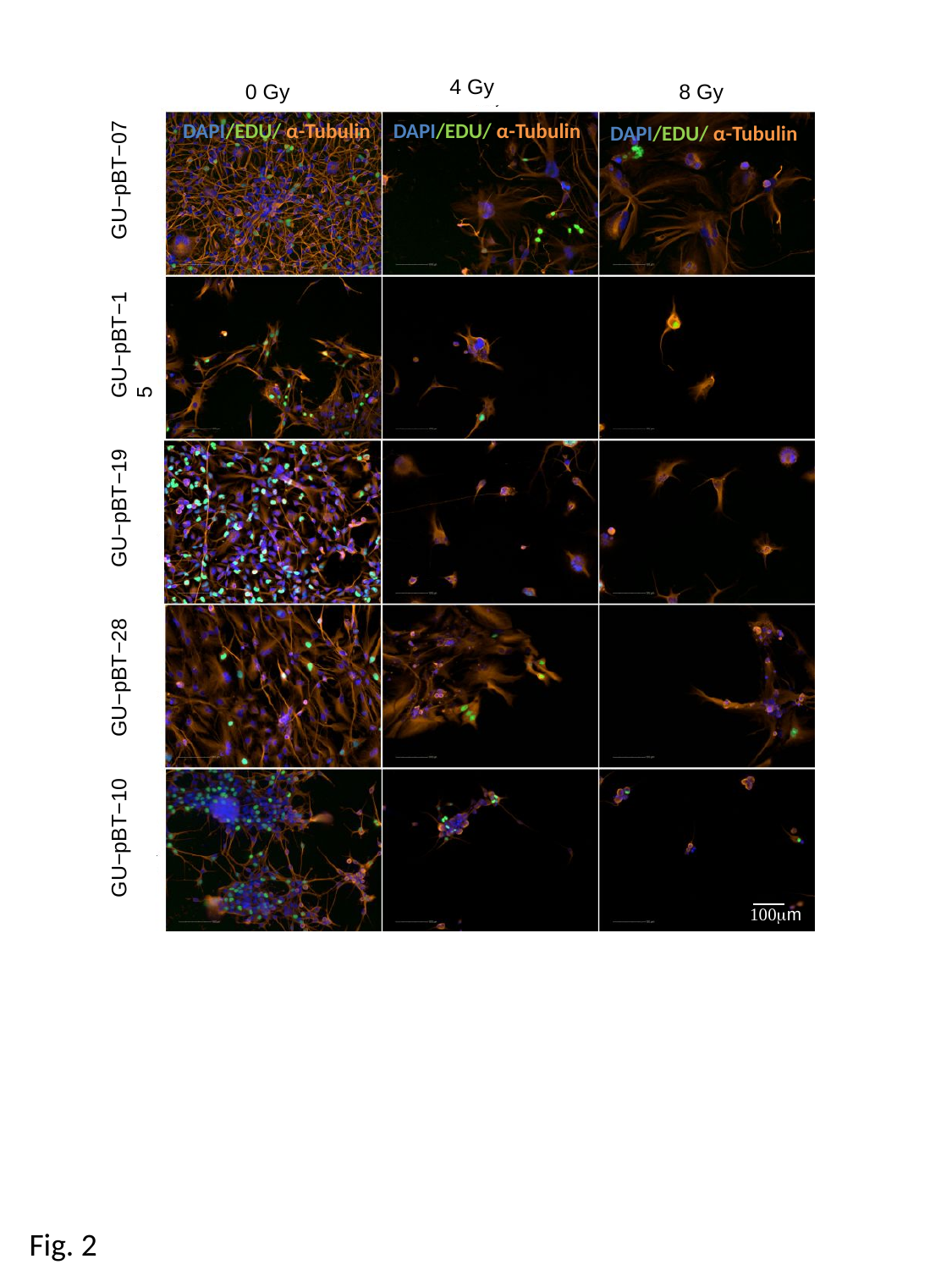

4 Gy
0 Gy
8 Gy
GU−pBT−07
DAPI/EDU/ α-Tubulin
DAPI/EDU/ α-Tubulin
GU−pBT−15
GU−pBT−19
GU−pBT−28
GU−pBT−10
100mm
DAPI/EDU/ α-Tubulin
Fig. 2

## Slide 3
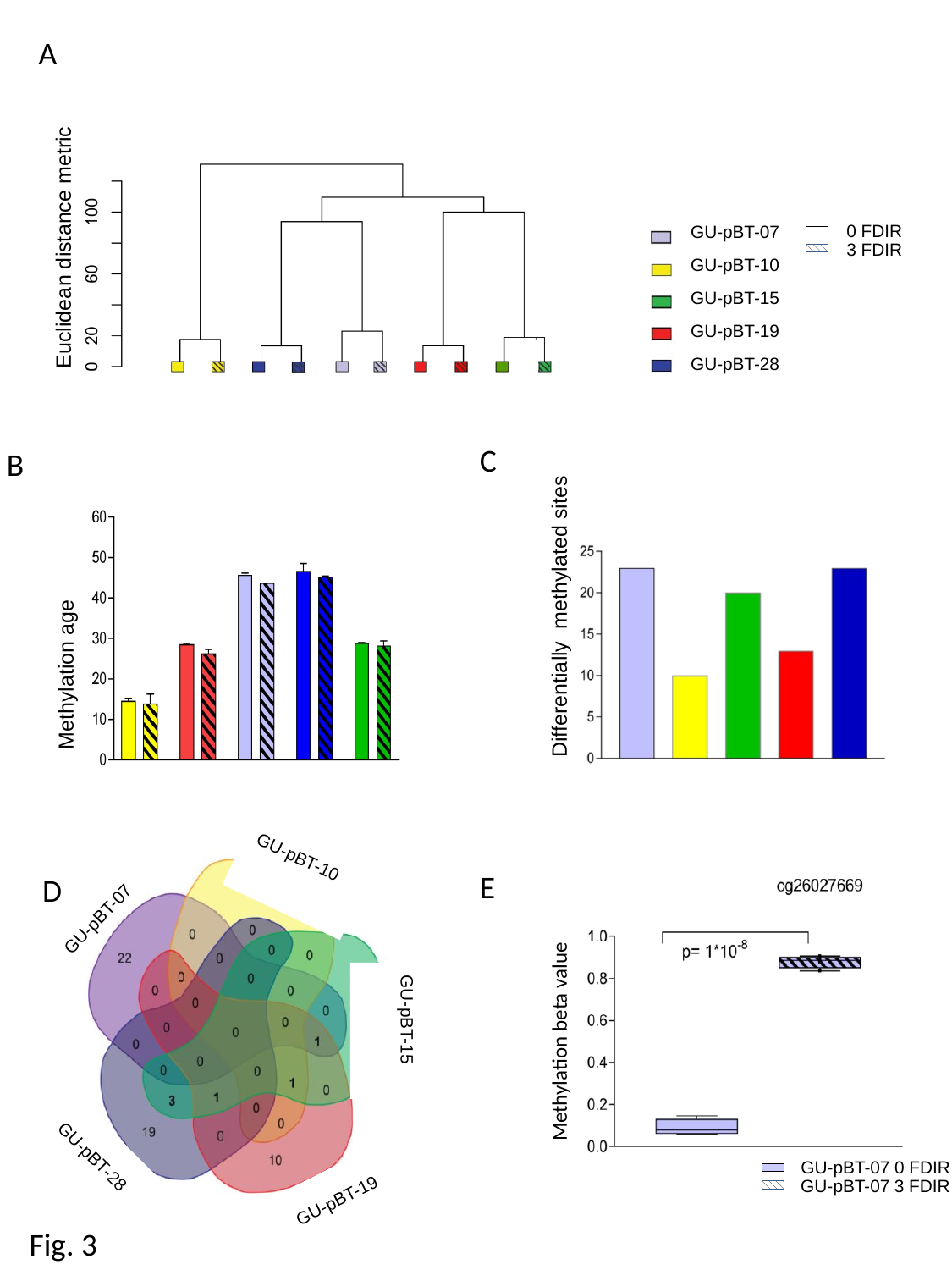

A
B
Methylation age
Euclidean distance metric
0 FDIR
3 FDIR
GU-pBT-07
GU-pBT-10
GU-pBT-15
GU-pBT-19
GU-pBT-28
C
E
Methylation beta value
Differentially methylated sites
GU-pBT-10
GU-pBT-07
GU-pBT-15
GU-pBT-28
GU-pBT-19
D
GU-pBT-07 0 FDIR
GU-pBT-07 3 FDIR
Fig. 3

## Slide 4
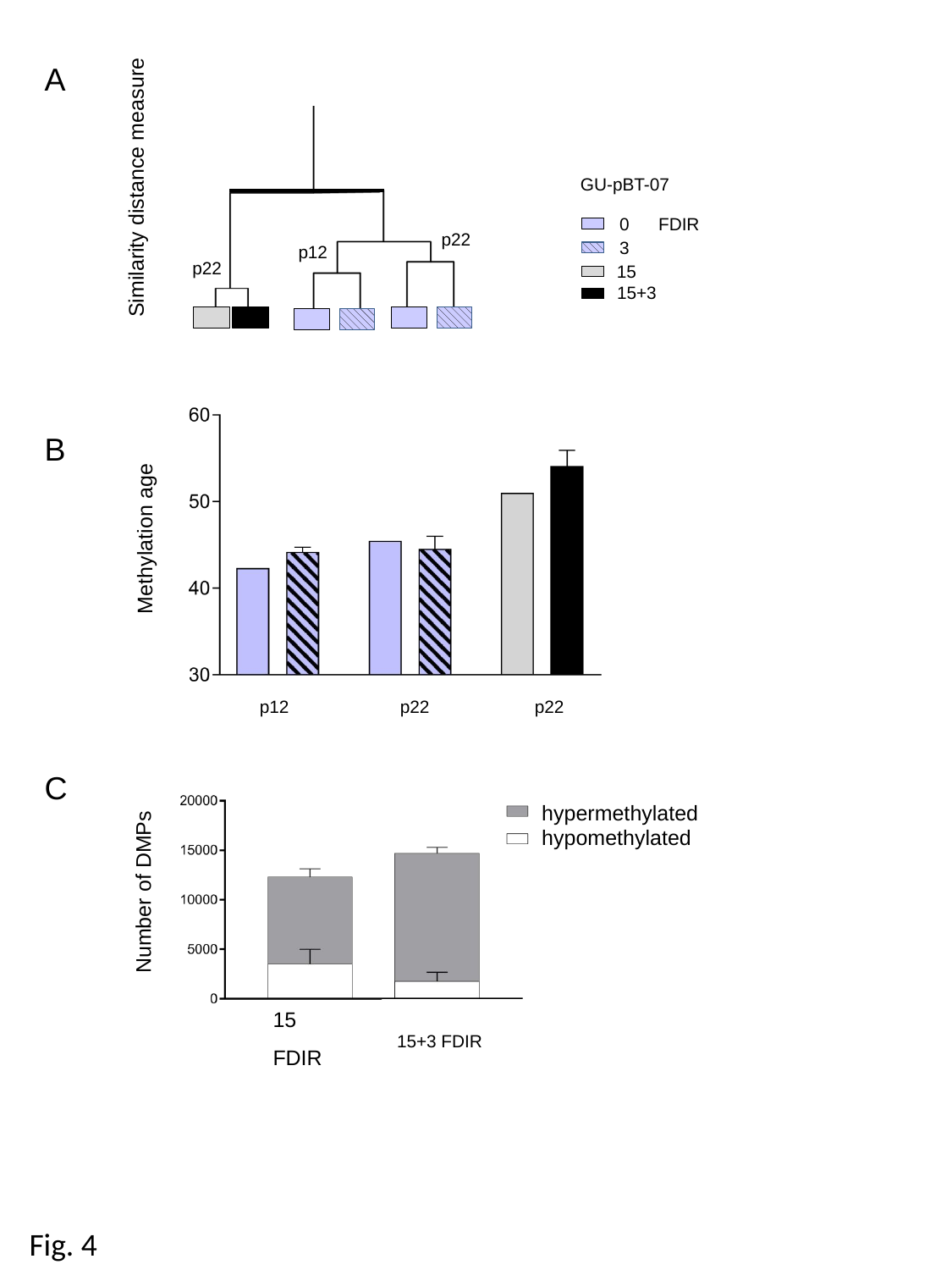

Similarity distance measure
A
0
3
15
15+3
GU-pBT-07
 FDIR
p22
p12
p22
B
Methylation age
p12
p22
p22
Number of DMPs
15+3 FDIR
15 FDIR
C
hypermethylated
hypomethylated
15+3 FDIR
18 FDIR
15 FDIR
Fig. 4

## Slide 5
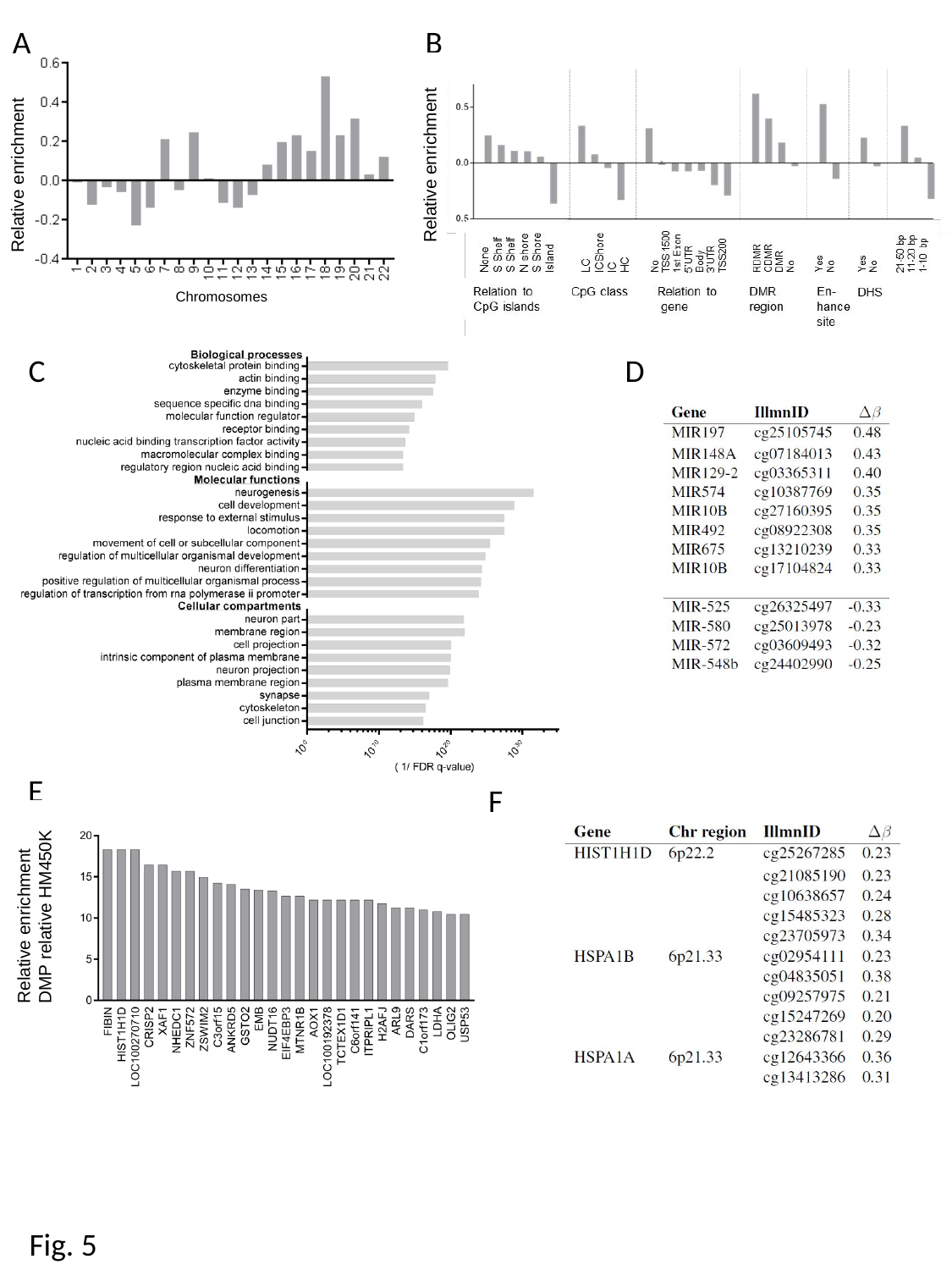

A
B
Relative enrichment
Relative enrichment
Chromosomes
C
D
E
F
Relative enrichment
DMP relative HM450K
Fig. 5

## Slide 6
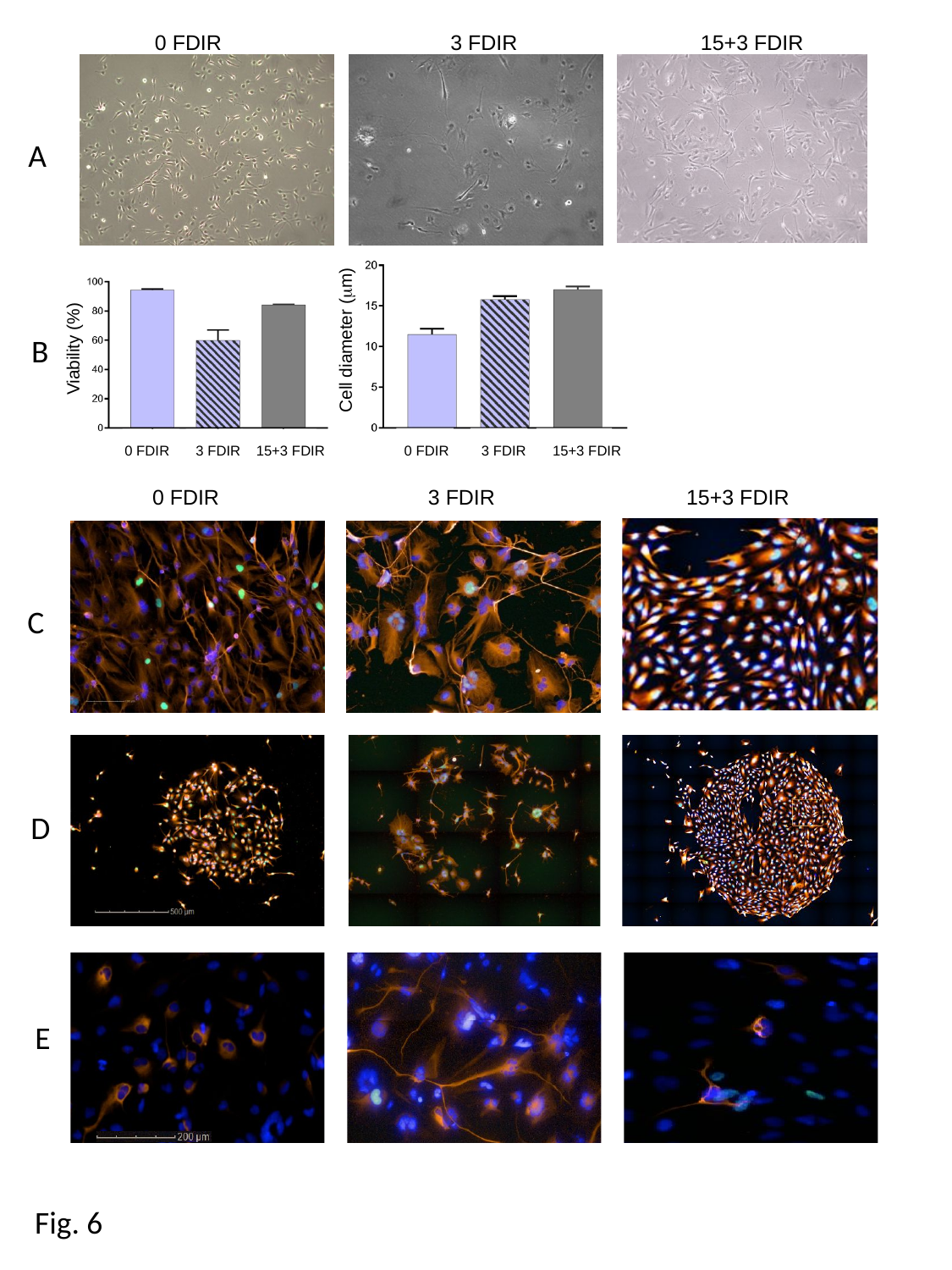

0 FDIR
3 FDIR
15+3 FDIR
A
B
Cell diameter (mm)
Viability (%)
0 FDIR
3 FDIR
15+3 FDIR
0 FDIR
3 FDIR
15+3 FDIR
0 FDIR
3 FDIR
15+3 FDIR
0 FDIR
3 FDIR
15+3 FDIR
0 FDIR
3 FDIR
15+3 FDIR
15 FDIR
C
D
0 FDIR
E
Fig. 6
